# Analysis of *GBE1* mutations via protein expression studies in glycogen storage disease type IV: A report on a non-progressive form with a literature review

**DOI:** 10.1016/j.ymgmr.2018.09.001

**Published:** 2018-09-13

**Authors:** Hiroyuki Iijima, Reiko Iwano, Yukichi Tanaka, Koji Muroya, Tokiko Fukuda, Hideo Sugie, Kenji Kurosawa, Masanori Adachi

**Affiliations:** a Department of Endocrinology and Metabolism, Kanagawa Children's Medical Center, Mutsukawa 2-138-4, Minami-ku, Yokohama 232-8555, Japan; b Department of Pathology, Kanagawa Children's Medical Center, Mutsukawa 2-138-4, Minami-ku, Yokohama 232-8555, Japan; c Department of Pediatrics, Hamamatsu University School of Medicine, Handayama, 1-20-1 Higashi-ku, Hamamatsu 431-3192, Japan; d Faculty of Health and Medical Sciences, Tokoha University, Sena, 1-22-1 Aoi-ku, Shizuoka 420-0911, Japan; e Division of Medical Genetics, Kanagawa Children's Medical Center, Mutsukawa 2-138-4, Minami-ku, Yokohama 232-8555, Japan

**Keywords:** Epilepsy, Functional analysis, Glycogen storage disease type IV, Glycogenosis, glycogen branching enzyme 1, GSD, APBD, adult polyglucosan body disease, GBE, 1,4-alpha-glucan-branching enzyme, GSD IV, glycogen storage disease type IV, NP-GSD IV, non-progressive form of glycogen storage disease type IV, RT-PCR, reverse transcriptase-polymerase chain reaction, WT, wild type

## Abstract

**Background:**

Glycogen storage disease type IV (GSD IV), caused by *GBE1* mutations, has a quite wide phenotypic variation. While the classic hepatic form and the perinatal/neonatal neuromuscular forms result in early mortality, milder manifestations include non-progressive form (NP-GSD IV) and adult polyglucosan body disease (APBD). Thus far, only one clinical case of a patient with compound heterozygous mutations has been reported for the molecular analysis of NP-GSD IV. This study aimed to elucidate the molecular basis in a NP-GSD IV patient via protein expression analysis and to obtain a clearer genotype-phenotype relationship in GSD IV.

**Case presentation:**

A Japanese boy presented hepatosplenomegaly at 2 years of age. Developmental delay, neurological symptoms, and cardiac dysfunction were not apparent. Observation of hepatocytes with periodic acid-Schiff-positive materials resistant to diastase, coupled with resolution of hepatosplenomegaly at 8 years of age, yielded a diagnosis of NP-GSD IV. Glycogen branching enzyme activity was decreased in erythrocytes. At 13 years of age, he developed epilepsy, which was successfully controlled by carbamazepine.

**Molecular analysis:**

In this study, we identified compound heterozygous *GBE1* mutations (p.Gln46Pro and p.Glu609Lys). The branching activities of the mutant proteins expressed using *E. coli* were examined in a reaction with starch. The result showed that both mutants had approximately 50% activity of the wild type protein.

**Conclusion:**

This is the second clinical report of a NP-GSD IV patient with a definite molecular elucidation. Based on the clinical and genotypic overlapping between NP-GSD IV and APBD, we suggest both are in a continuum.

## 1 Introduction

Glycogen storage disease type IV (GSD IV; Andersen disease [1]; OMIM #232500) is a rare autosomal recessive metabolic disorder caused by a deficiency of amylo-(1,4 to 1,6)-transglucosidase (EC 2.4.1.18, 1,4-alpha-glucan-branching enzyme, GBE). It is characterized by the accumulation of an amylopectin-like glycogen (polyglucosan) in multiple organs, such as the liver, muscle, heart, and the central and peripheral nervous systems [2]. Several phenotypic categories have been reported for GSD IV [3]. The classic hepatic form is the most common, wherein patients progress rapidly to cirrhosis and tend to die no later than 5 years of age, unless liver transplantation is attempted [1,[4], [5], [6], [7], [8]]. Meanwhile, patients with the non-progressive form (NP-GSD IV) display hepatosplenomegaly and elevated transaminase levels, which regress spontaneously without any features of cirrhotic, neurologic, muscular, or cardiac involvement [4,9]. In addition, a neuromuscular form has been reported, which is further sub-divided in accordance with the age at onset (perinatal, neonatal, juvenile, or adult). Patients with the perinatal form present in utero fetal akinesia deformation sequence, polyhydramnios, fetal hydrops, arthrogryposis, and perinatal death [5,[10], [11], [12], [13], [14], [15], [16], [17], [18], [19]]. Those with the neonatal form present hypotonia, muscular atrophy, and cardiomyopathy immediately postpartum, and usually die in the neonatal period [[4], [5], [6],^16^,^18^,[20], [21], [22], [23], [24]]. The juvenile form is dominated by myopathy or cardiomyopathy with pubertal or young adult onset [5,25]. The adult form, also known as adult polyglucosan body disease (APBD), is characterized by adult-onset multisystem disorder including myopathy or neurological involvement such as neurogenic bladder, seizure, or spastic paraplegia with vibration loss and numbness [[26], [27], [28], [29], [30], [31], [32]].

**Fig. 1:**
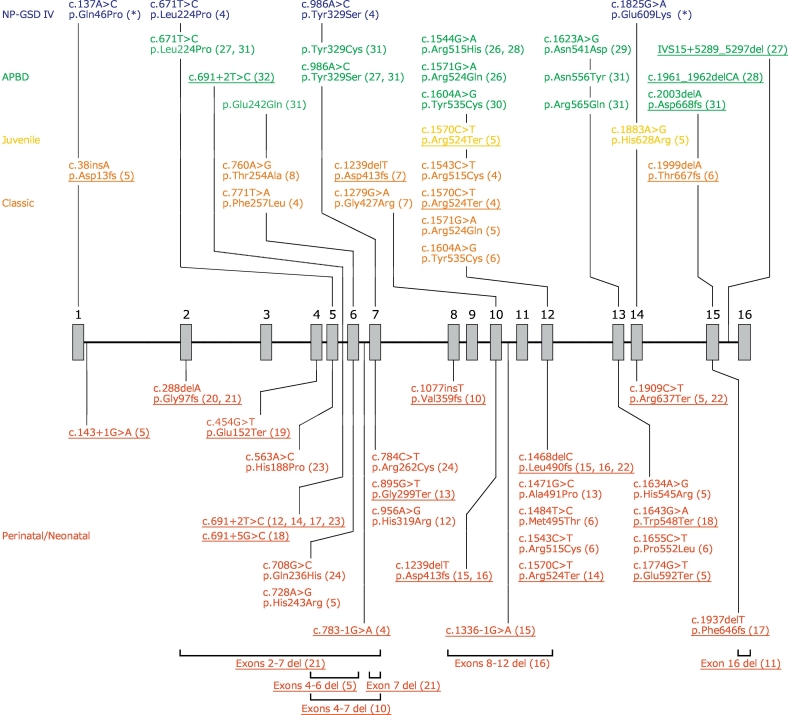
Organization of the *GBE1* gene, and disease-associated mutations hitherto reported. The number above each box indicates the exon number. References are denoted in parentheses. The mutations identified in our patient are indicated by an asterisk. Null mutations such as intragenic deleterious, nonsense, frameshift, and splice-site mutations are underlined. Herein, we gathered neonatal and perinatal forms in a mass because their diagnostic criteria are not strictly determined and clinical outcomes in these forms do not differ significantly. Null mutations, except for those located in exons 15 and 16, tend to associate with more severe forms of glycogen storage disease type IV (GSD IV), such as classic hepatic form or perinatal/neonatal neuromuscular forms. The same mutations are often reported in unrelated patients with milder forms, such as non-progressive-GSD IV (NP-GSD IV) and adult polyglucosan body disease (APBD).

The aforementioned various manifestations in GSD IV result from mutations in a single responsible gene, *GBE1* (*607839). *GBE1* is located on chromosome 3p14, consists of 16 exons, and encodes a protein of 702 amino acid residues [33]. Thus far, 52 different *GBE1* mutations have been reported, including missense, nonsense, deleterious, insertional, and splice-site mutations (Fig. 1). To our knowledge, however, only two mutations (p.Leu224Pro and p.Tyr329Ser) in a single patient have been identified in NP-GSD IV [4]. Interestingly, one of these mutations (p.Tyr329Ser) is a founder mutation of APBD among individuals of Ashkenazi-Jewish descent [27,31]. In addition, functional protein expression analysis has been performed in only one study [4].

Herein, we describe a case of NP-GSD IV caused by novel missense *GBE1* mutations and present a review of the literature to obtain a clearer genotype-phenotype relationship in GSD IV.

## 2 Case report

**Fig. 2:**
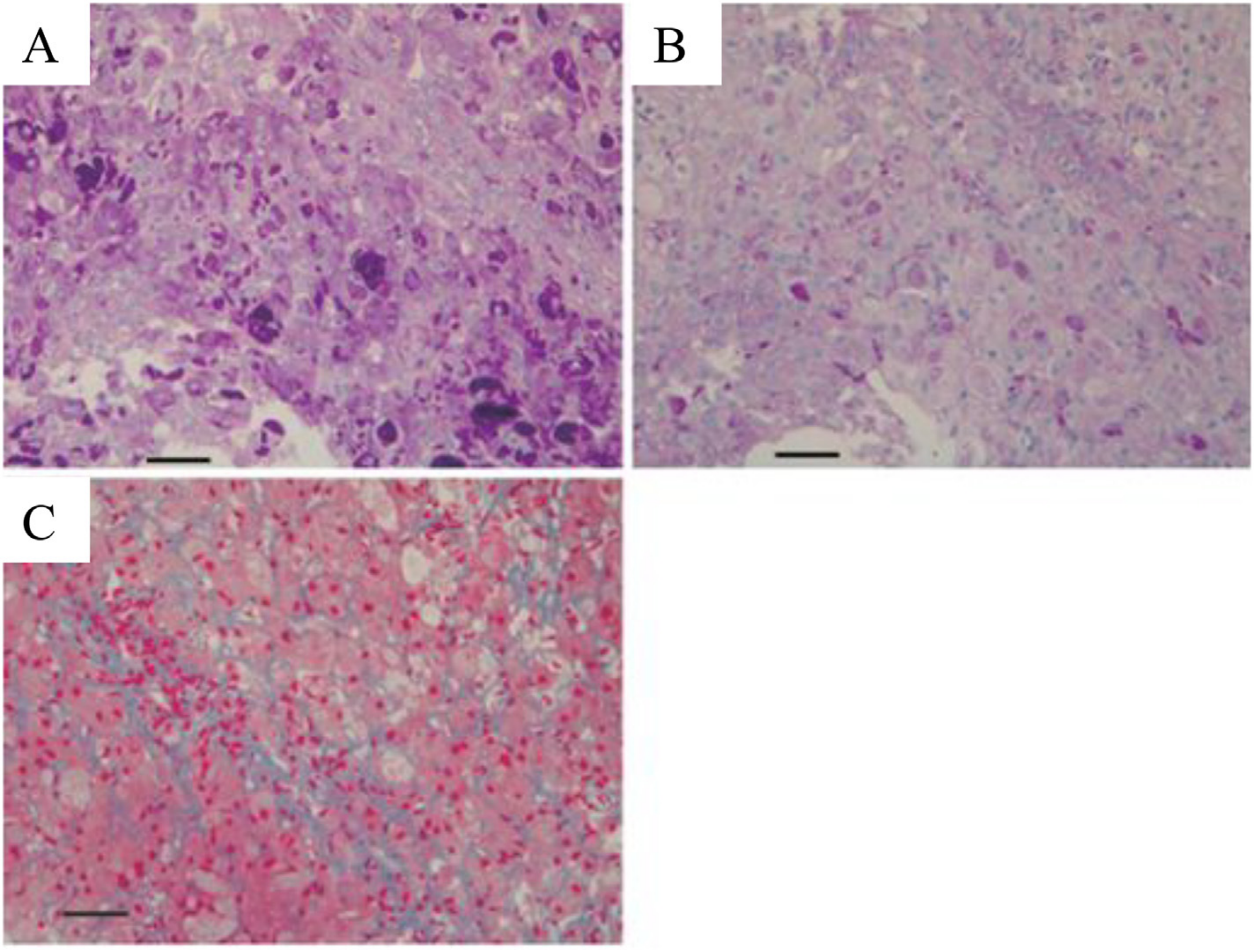
Light microscopic images from the liver biopsy specimen of our patient (bar: 100 μm). The hepatocyte cytoplasm was stained with periodic acid-Schiff (PAS) stain with a coarse granular pattern (A). PAS-positive deposits were partially diastase-resistant (B). A mild fibrotic change was observed via Azan staining (C).

A Japanese boy was referred to our hospital at 2 years of age because of elevated serum transaminases. He was born after an uneventful pregnancy and had no significant family history. At the time of admission, his height and weight were 80.9 cm (−1.5 SD) and 10.54 kg (−0.9 SD), respectively. He had hepatosplenomegaly (liver 6 cm below the right costal margin and spleen 1.5 cm below the left costal margin). His developmental milestones were normal, and he did not present any neurologic symptoms. Laboratory data indicated an elevation of serum transaminases (AST 221 IU/l and ALT 124 IU/l), without any other metabolic derangements such as hypoglycemia, hyperlipidemia, hyperuricemia, or hyperlactacidemia. Viral hepatitis and autoimmune hepatitis were denied upon appropriate laboratory tests. In the oral glucose tolerance test (2.5 g/kg), an adequate increase in blood lactate levels was observed (fasting, 10.3 mg/dl; 60 min, 19.0 mg/dl, and 120 min, 16.7 mg/dl). Glucagon stimulation test (0.03 mg/kg) on fasting yielded a significant response regarding blood glucose, with 74 mg/dl before, and 113 mg/dl 30 min after stimulation. Upon liver biopsy, periodic acid-Schiff-positive cytoplasmic inclusions were reported with partial resistance to diastase digestion (Fig. 2), which yielded a diagnosis of GSD IV. There were, however, no histological changes or laboratory data indicating cirrhosis. Moreover, serum transaminase levels normalized at 5 years of age, followed by a disappearance of hepatosplenomegaly at 8 years of age. Cardiac function was adequate, as verified via ultrasonic cardiography, and he did not present any myopathic symptoms. However, he developed epilepsy at 13 years of age and was administered carbamazepine therapy. There were no abnormal findings in brain magnetic resonance image taken at 14 years of age. At present, he is 17 years old and has normal liver function, without discernible hepatosplenomegaly. Except for favorably controlled epilepsy, he has not shown any neurological symptoms, such as ataxia, muscle weakness, gait disturbance and urinary retention. He is now studying for college entrance examination.

## 3 Materials and methods

Written informed consent was obtained from the patient and his parents for all experiments described herein, per the tenets of the Declaration of Helsinki, in addition to consent for publication.

### 3.1 Analysis of GBE activity

Erythrocyte GBE activity was analyzed using a previously described method [34,35]. Phosphorylase b kinase enzyme activity in erythrocytes was measured to eliminate the possibility of sample inactivation.

### 3.2 *GBE1* mutation analysis

*GBE1* mutation analysis was performed in gDNA extracted from peripheral blood leukocytes of the patient and his parents. Direct Sanger sequencing of the *GBE1* gene was performed after amplification of all 16 exons and intron/exon boundaries via PCR with an Applied Biosystems® Veriti Thermal Cycler (Thermo Fisher Scientific, MA, USA) in accordance with a previously described method [22]. Next-generation sequencing was performed using MiSeq® bench-top sequencer (Illumina, San Diego, CA, USA) with a TruSight® One Sequencing Panel (Illumina). Common genetic variations were identified using our in-house and public databases (dbSNP, 1000 Genomes Project, NHLBI Exome Sequencing Project, Human Genetic Variation Database (HGVD), NCBI ClinVar and Human Gene Mutation Database).

### 3.3 Reverse transcriptase-PCR (RT-PCR)

*GBE1* sequence was obtained from GenBank (NG_011810), which was then applied to the human genome assembly (http://genome.ucsc.edu) using BLAT to identify exon/intron boundaries. Gene-specific PCR primers were designed using Primer3 (http://bioinfo.ut.ee/primer3-0.4.0/) and NCBI primer BLAST software (http://www.ncbi.nlm.nih.gov/tools/primer-blast/) (Supplemental Table 1). Total RNA was extracted from leukocytes, using the ISOGEN II® reagent (FUJIFILM Wako Pure Chemical Corporation, Osaka, Japan). Total RNA was eluted in a final volume of 20 μl of RNase-free water and stored at −80 °C until use. RT-PCR was performed with the PrimeScript® One Step RT-PCR Kit (Takara Bio Inc., Shiga, Japan) in accordance with the manufacturer's instructions.

### 3.4 In silico analysis

Phylogenic information was obtained via Vertebrate Multiz Alignment & Conservation in UCSC Genome Browser (https://genome.ucsc.edu/). We used PolyPhen-2 (http://genetics.bwh.harvard.edu/pph2/) and SIFT (http://asia.ensembl.org/index.html) predictive algorithms to evaluate the pathogenicity of the identified sequence variants. PyMOL software (https://pymol.org/2/) was used to speculate potential conformational changes in mutant GBE1.

### 3.5 Functional analysis of mutant GBE proteins

To evaluate the pathogenicity of the sequence variations in *GBE1* identified in the patient, functional analysis with in vitro expression experiments was conducted as follows. We used *E. coli* BL21 (DE3) cells (Merck, Darmstadt, Germany) to express wild type (WT) and mutant GBE proteins. Branching activity of each protein was then measured by the previously described method that utilizes amylose‑iodine absorbance spectrum [37]. Because amylose, which constitutes starch with amylopectin, has a linear structure, GBE can act on amylose to form branching points even in the absence of other enzymes required for glucose-chain elongation. Once branched, amylose will fail to develop amylose‑iodine specific absorbance spectrum at 660 nm (A660). Accordingly, by measuring the reduction of A660 by spectrometry, GBE activity can be estimated.

#### 3.5.1 Construction of WT and mutant *GBE1* cDNAs

To construct cDNA sequences containing either p.Gln46Pro or p.Glu609Lys mutations, site-directed mutagenesis was performed using pET-21b vector Novagen (Merck). Each cDNA, including WT, was artificially synthesized and then inserted into the *Nde*I/*Xho*I sites of the vector. Stop codons were eliminated and polyhistidine-tags were inserted at the c-termini.

#### 3.5.2 Protein expression, purification, and dialysis

WT and mutant cDNA in vector pET-21b were transfected into BL21 (DE3) cells (Merck). After incubation at 37 °C overnight, each colony was inoculated into Luria-Bertani (LB) liquid medium (supplemented with 100 μg/ml Ampicillin) and incubated at 37 °C with agitation (220 rpm) until the OD_600_ approached 0.6–1.0. Isopropyl β-D-1-thiogalactopyranoside was added into the culture to induce target protein expression. After cell lysis by ultra-sonication and centrifugation at 12,000 rpm for 15 min (low temperature), the supernatant was loaded onto a Ni NTA affinity column (Thermo Fisher Scientific) pre-equilibrated with lysis buffer (50 mM NaH2PO4, 300 mM NaCl, and 20 mM imidazole). The column was then washed with wash Buffer (50 mM NaH2PO4, 300 mM NaCl, and 50–100 mM imidazole) to elute the irrelevant proteins, until the OD_280_ of the eluent approached baseline values. The eluent containing 100 mM imidazole was dialyzed with PBS buffer and analyzed via sodium dodecyl sulfate polyacrylamide gel electrophoresis (SDS-PAGE; 12% resolving gel).

#### 3.5.3 Spectrophotometric analysis of enzyme activity

The present assay employed starch as the substrate, which would be branched by GBE. After a certain incubation period, amylose content can be determined by staining with iodine. GBE activity was determined when the initial reaction velocity appeared linear.

The reaction mixture contained 6 mg/ml starch in 10 mM sodium phosphate buffer (pH 7.4), containing 100 mM sodium chloride. The reaction mixture (0.05 ml) was mixed with 1.0 ml iodine reagent (0.5 mg/ml iodine and 5 mg/ml potassium iodide in water). A positive correlation was confirmed between the spectrophotometric absorbance at 660 nm (A660) and the starch concentration in the reaction mixture. To determine a suitable reaction time point, ΔA660 (A660 at 30 min minus the A660 at 0 min) was observed after the addition of standard GBE enzyme, which indicated that the time point of 30 min was suitable.

Thereafter, the activity of each enzyme, namely, WT-GBE, Gln46Pro-GBE, and Glu609Lys-GBE, was analyzed as follows. The reaction mixture comprised 0.25–0.33 mg/ml of GBE enzyme, 6 mg/ml starch in 10 mM sodium phosphate buffer (pH 7.4), containing 100 mM sodium chloride, where 0.05 ml of the reaction mixture was mixed with 1.0 ml iodine reagent prior to spectrophotometric measurement of A660. Each enzyme assay was performed in duplicate. One unit of GBE activity was defined as the amount of the decrease of 1 mg starch per minute at 37 °C during 30 min. In other words,

$$\text{Activity}\;\left(U/\mathrm{mg}\right)=\mathrm{\Delta A}660/S/T/C/V$$

S: slope of the standard curve, T: reaction time (30 min), C: concentration of the GBE protein, V: volume of the GBE protein (0.001 ml).

## 4 Results

### 4.1 Analysis of GBE activity

Erythrocyte GBE activity in the patient was lower (0.3 μmol Pi/min/g Hb) than that of the three control samples (2.4, 2.5, and 3.2 μmol Pi/min/g Hb). Positive disease control samples displayed a GBE activity of 0.2 μmol Pi/min/g Hb. Erythrocyte phosphorylase b kinase activity of the patient was normal.

### 4.2 *GBE1* mutation analysis

**Fig. 3:**
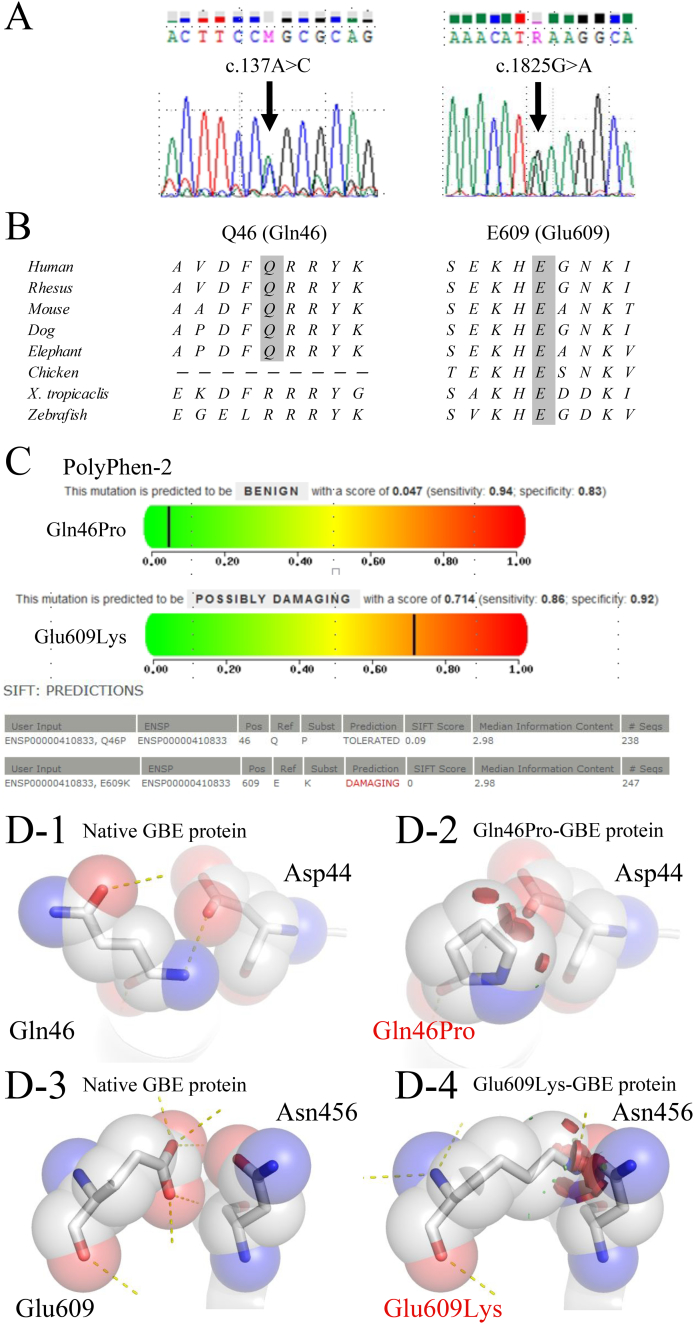
*GBE1* mutation analysis. Electropherogram of *GBE1* gDNA of our patient, showing heterozygous missense mutations (arrow). (B) Glutamine at position 46 (Gln46) and glutamate at position 609 (Glu609) in *Homo sapiens GBE1* are conserved. (C) PolyPhen-2 and SIFT analyses of the p.Gln46Pro and p.Glu609Lys mutations. (D) A structural model of GBE protein generated via PyMOL. Hydrogen bonding is indicated with dashed lines and steric hindrance is indicated with discs. (D-1, D-3) The native GBE structure shows that the Gln46 and Glu609 interact with Asp44 and Asn456, respectively. (D-2, D-4) The modeled structure of the mutant proteins (Gln46Pro-GBE and Glu609Lys-GBE) is speculated to have structural changes caused by steric hindrance with Asp44 and Asn456, respectively.

Sanger sequencing of *GBE1* revealed heterozygous missense mutations (c.137A > C [p.Gln46Pro, located in exon 1] and c.1825G > A [p.Glu609Lys, located in exon 14]) in the patient (Fig. 3A). The former mutation was detected in his father, whereas the latter mutation was detected in his mother, both being heterozygous mutations. A homozygous c.568A > G substitution was detected in the patient and in his father and was reported in 19% of 1207 healthy controls from the HGVD. This substitution was listed in the dbSNP database (rs2229519). Next-generation sequencing revealed the same mutations (p.Gln46Pro and p.Glu609Lys) in *GBE1*. According to HGVD, p.Gln46Pro was not listed, whereas p.Glu609Lys was listed with a frequency of 0.00206782. In addition, p.Glu609Lys was listed as rs772802187 in dbSNP.

### 4.3 RT-PCR

No aberrant splice variant was identified (data not shown). Direct sequencing of RT-PCR products revealed the same mutations (p.Gln46Pro and p.Glu609Lys).

### 4.4 In silico analysis

Both Gln46 and Glu609 are conserved throughout species (Fig. 3B). Upon PolyPhen-2 and SIFT analysis, p.Gln46Pro was predicted as “not damaging”. However, PolyPhen-2 and SIFT analyses predicted p.Glu609Lys to be “possibly damaging” and “damaging,” respectively (Fig. 3C). The modeled structure of Gln46Pro-GBE and Glu609Lys-GBE are shown in Fig. 3D. In both GBE proteins, the mutation was speculated to disrupt hydrogen bonds on the molecular circumference and to cause steric hindrance with Asp44 and Asn456, respectively (Fig. 3D).

### 4.5 Functional analysis of mutant GBE proteins

The enzyme activity of Gln46Pro-GBE and Glu609Lys-GBE was low (1.21 and 1.15 U/mg, respectively) compared with that of WT-GBE (2.18 U/mg), corresponding to 56% and 53% of WT-GBE activity, respectively (Table 1).

**Table 1:** Functional analysis of mutant GBE proteins.

	Concentration (mg/dl)	A660 average (range)	ΔA660 average	GBE activity (U/mg)
0 min	0	0.998 (0.986–1.01)	–	–
WT-GBE (30 min)	0.25	0.710 (0.705–0.714)	0.289	2.18
Gln46Pro-GBE (30 min)	0.37	0.762 (0.759–0.764)	0.237	1.21
Glu609Lys-GBE (30 min)	0.33	0.796 (0.775–0.817)	0.202	1.15

Each assay was performed in duplicate. Activity (U/mg) = ΔA660/S/T/C/V, where S is the slope of the standard curve; T, reaction time (30 min); C, concentration of GBE protein; V, volume of the GBE protein added (0.01 ml). One unit is defined as the amount of the decrease of 1 mg starch per minute at 37 °C in the reaction mixture. A660 is the spectrophotometric absorbance at 660 nm; ΔA660 is the A660 at 30 min minus the A660 at 0 min.

## 5 Discussion

Two novel missense mutations, p.Gln46Pro and p.Glu609Lys, were identified in a compound heterozygous state, in a potentially recessive pattern of inheritance. To our knowledge, this is the second report wherein enzymatic activity of mutant GBE proteins was verified on the basis of molecular expression analyses in any form of GSD IV. The present results indicate that Gln46Pro-GBE retained 56% and Glu609Lys-GBE 53% residual activity compared to WT-GBE, which may argue against the pathogenicity of the mutations. However, the only previous study based on molecular expression analysis reported that the activity of Tyr329Ser-GBE and Leu224Pro-GBE, responsible for NP-GSD IV, were 54% and 8.7% of WT-GBE, respectively [4]. In addition, most APBD patients of Ashkenazi-Jewish descent have a homozygous p.Tyr329Ser mutation [27,31]. Therefore, we assume that approximately 50% of GBE activity is compatible with the milder forms of GSD IV, namely, NP-GSD IV and APBD. PyMOL analysis suggested significant structural abnormality (Fig. 3D), similar to phylogenic analysis (Fig. 3B), which may strengthen the pathogenicity of the mutations. In silico prediction models failed to reveal the damaging nature of p.Gln46Pro (Fig. 3C). However, these results can be considered to indicate residual activity. Thus, we concluded that both p.Gln46Pro and p.Glu609Lys mutations are truly pathogenic.

A previous study reported a suitable genotype-phenotype relationship in GSD-IV (Fig. 1). All null mutations, underlined in Fig. 1, have resulted in more severe forms including classic hepatic and perinatal/neonatal neuromuscular forms, in the homozygous state. Interestingly, null mutations in exons 15 and 16 tend to lead to milder forms, namely, NP-GSD IV and APBD probably because the central (β/α) barrel catalytic domain of GBE encompassing amino acid residues 184 to 600 having catalytic capacity correspond to exons 4–13 [36]. In contrast, although missense mutations tend to associate with milder forms (NP-GSD IV and APBD), identical mutations are often reported in unrelated patients with both forms [4,27,31]. Accordingly, a genotype-phenotype relationship is less clear in the milder phenotypes of GSD-IV.

To clarify this ambiguity, we suggest that NP-GSD IV and APBD are overlapping each other. Paradas et al. reported a case of GSD IV (homozygote of p.Arg515His mutation in *GBE1*), which was compatible with both NP-GSD IV and APBD [28]. This female patient had undergone liver biopsy at 2 years of age because of hepatomegaly and elevated transaminase levels, which revealed glycogen accumulation. Following a long latent period without any hepatic manifestations, she suddenly developed neurological symptoms at 44 years of age, such as difficulty in writing and walking. In another case reported by Mochel et al.*,* typical APBD in the early 30's was initially reported as NP-GSD IV [9,31]. In addition, compound heterozygous mutations (p.Leu224Pro and p.Tyr329Ser) in *GBE1* were identified in both NP-GSD IV and APBD. Therefore, we advocate NP-GSD IV and APBD are in a continuum, and long-term surveillance for neurological symptoms is necessary for the patients with NP-GSD IV.

Clinical profile of the patient reported in the current study, namely, transient nature of hepatosplenomegaly and elevated transaminase levels, in the absence of any cardiac and myopathic complications, is typical for NP-GSD IV. To our knowledge, no previous report has indicated the development of epilepsy in NP-GSD IV individuals. In addition, the patient has never developed neurological symptoms, except for epilepsy, until 17 years of age. Accordingly, an occurrence of epilepsy in the patient seems not to be an early sign of APBD, but may be fortuitous. Longer observation is mandatory to elucidate this association.

There are two limitations in our method of functional analysis. First, we used *E. coli* BL21 (DE3) strain for expression analysis, and not a mammalian one such as HEK293 cells, mainly because of economical constraints. However, our results need to be verified using mammalian cell expression system. Second, we used starch as a substrate of mutant and WT GBE, not glycogen, for the simplicity of methodology. If glycogen were used as a substrate, 1) enzymes for glucose-chain elongation, such as glycogen synthase, must be added in the assay system, and 2) amylose‑iodine absorbance spectrum is not available. However, because starch is not naturally present in the human body, it would be ideal to measure GBE activity by using glycogen as substrate.

## 6 Conclusion

The present results indicate novel *GBE1* mutations (p.Gln46Pro and p.Glu609Lys) in a NP-GSD IV patient. This is, to our knowledge, the second report on the molecular basis of NP-GSD IV. Furthermore, only one previous study verified the pathogenicity of *GBE1* mutations via analysis of molecular expression. The concept of NP-GSD IV and APBD being in a continuum will further clarify the genotype-phenotype relationship in GSD IV, for which a long-term follow-up is necessary.

The following is the supplementary data related to this article.

Supplementary Table S1 Primers for reverse-transcription polymerase chain reaction and cycling conditions. Supplementary Table S1

## Conflict of interest

The authors declared no potential conflicts of interest with respect to the research, authorship, and/or publication of this article.
