# Supplementary material for: Analysis of GBE1 mutations via protein expression studies in glycogen storage disease type IV: A report on a non-progressive form with a literature review
Source: Mol Genet Metab Rep. 2018 Sep 13;17:31–7. doi: 10.1016/j.ymgmr.2018.09.001 (PMC6140619; doi:10.1016/j.ymgmr.2018.09.001)
Supplement: Supplementary Table S1 — Primers for reverse-transcription polymerase chain reaction and cycling conditions. [file mmc1.docx]

Supplemental Table 1. Primers for reverse-transcription polymerase chain reaction and cycling conditions

| Primer name | | Sequence (5'-3') | Annealing temperature | Amplicon length |
| --- | --- | --- | --- | --- |
|  |  |  |  |  |
| Exon 1-3 | F | TCGGCTACTTGGACTGCG | 60.4°C | 481 bp |
|  | R | CTCTCCGCTTTTACTAGTAATAACTACCTT | 61.0°C |  |
| Exon 3-7 | F | GCAGAATAAATCTGTACTCGTGCC | 60.4°C | 527 bp |
|  | R | CATCAAACATATTCAATCCATCTGCTG | 59.7°C |  |
| Exon 7-9 | F | GATGTGGTACACAGCCATGC | 60.0°C | 396 bp |
|  | R | CATTCCTGATACATCCTCAGCTATTG | 59.9°C |  |
| Exon 9-13 | F | TCACACGCTGTGTCCCGATTC | 63.7°C | 465 bp |
|  | R | CCTTTTCTTGGGAAGTCTAACCATTCAG | 62.6°C |  |
| Exon 12-16 | F | GCTTGGTGGAGAAGGCTATCTC | 61.3°C | 566 bp |
|  | R | CAAAACACAAATCTGCATCTGGTGG | 62.0°C |  |
